# 2-[(4-Chloro­phenyl)­amino­meth­yl]phenol

**DOI:** 10.1107/S1600536811032557

**Published:** 2011-08-17

**Authors:** Jie Xu

**Affiliations:** a Ordered Matter Science Research Center, College of Chemistry and Chemical Engineering, Southeast University, Nanjing 210096, People’s Republic of China

## Abstract

In the title mol­ecule, C_13_H_12_ClNO, the two benzene rings are twisted from each other by a dihedral angle of 68.60 (8)°. In the crystal structure, the hy­droxy and amino H atoms are involved in inter­molecular hydrogen bonds, O—H⋯N and N—H⋯O, respectively, resulting in *R* ^4^_4_(8) loops about inversion centers.

## Related literature

For the properties and structures of related amino compounds, see: Fu *et al.* (2007 ▶, 2008 ▶, 2009 ▶); Fu & Xiong (2008 ▶). For a description of ring motifs, see: Bernstein *et al.* (1995 ▶).
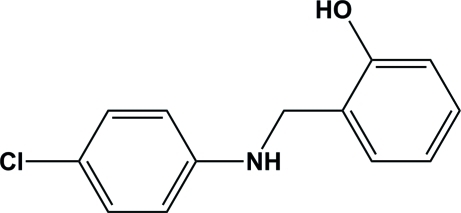


## Experimental

### 

#### Crystal data

C_13_H_12_ClNO
*M* *_r_* = 233.69
Triclinic, 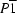

*a* = 5.5842 (11) Å
*b* = 7.9485 (16) Å
*c* = 13.023 (3) Å
α = 86.87 (3)°
β = 89.12 (3)°
γ = 88.65 (3)°
*V* = 577.0 (2) Å^3^
*Z* = 2
Mo *K*α radiation
μ = 0.31 mm^−1^
*T* = 298 K
0.10 × 0.03 × 0.03 mm

#### Data collection

Mercury2 (2×2 bin mode) diffractometer
Absorption correction: multi-scan (*CrystalClear*; Rigaku, 2005 ▶) *T*_min_ = 0.910, *T*_max_ = 1.000
5968 measured reflections
2637 independent reflections
1383 reflections with *I* > 2σ(*I*)
*R*_int_ = 0.061

#### Refinement

*R*[*F* ^2^ > 2σ(*F* ^2^)] = 0.068
*wR*(*F* ^2^) = 0.187
*S* = 0.98
2637 reflections
145 parameters
H-atom parameters constrained
Δρ_max_ = 0.22 e Å^−3^
Δρ_min_ = −0.25 e Å^−3^

### 

Data collection: *CrystalClear* (Rigaku, 2005 ▶); cell refinement: *CrystalClear*; data reduction: *CrystalClear*; program(s) used to solve structure: *SHELXS97* (Sheldrick, 2008 ▶); program(s) used to refine structure: *SHELXL97* (Sheldrick, 2008 ▶); molecular graphics: *SHELXTL* (Sheldrick, 2008 ▶); software used to prepare material for publication: *SHELXTL*.

## Supplementary Material

Crystal structure: contains datablock(s) I, global. DOI: 10.1107/S1600536811032557/pv2434sup1.cif

Structure factors: contains datablock(s) I. DOI: 10.1107/S1600536811032557/pv2434Isup2.hkl

Supplementary material file. DOI: 10.1107/S1600536811032557/pv2434Isup3.cml

Additional supplementary materials:  crystallographic information; 3D view; checkCIF report

## Figures and Tables

**Table 1:** Hydrogen-bond geometry (Å, °)

*D*—H⋯*A*	*D*—H	H⋯*A*	*D*⋯*A*	*D*—H⋯*A*
O1—H1⋯N1^i^	0.82	1.97	2.780 (3)	171
N1—H1*A*⋯O1^ii^	0.89	2.17	3.037 (3)	165

Symmetry codes: (i) 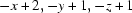; (ii) 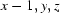.

**Fig. 1.**
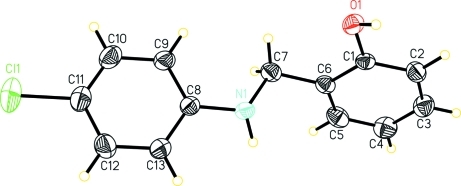
A view of the title compound with the atomic numbering scheme. Displacement ellipsoids are drawn at the 30% probability level. H atoms are represented as small spheres of arbitrary radii.

**Fig. 2.**
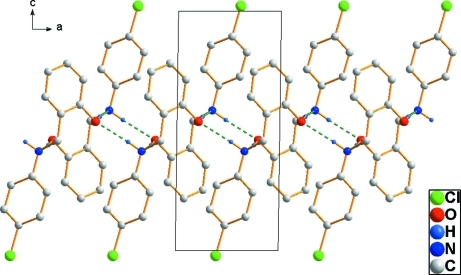
The crystal packing of the title compound, showing the one-dimensional chain. H atoms not involved in hydrogen bonding (dashed line) have been omitted for clarity.

## supplementary crystallographic information

### Comment

Organic amino compounds have attracted much attention as phase transition dielectric materials for their application in memory storage (Fu *et al.*, 2007; Fu & Xiong 2008; Fu *et al.*, 2008; Fu *et al.*, 2009). With the purpose of obtaining phase transition crystals of amino compounds, various amines have been studied and we have developed a series of new organic molecules. In this article, we describe the crystal structure of the title compound.

In the title molecule (Fig. 1), the two benzene rings are twisted from each other by a dihedral angle of 68.60 (8)°. In the crystal structure, the hydroxyl and amino H atoms are involved in intermolecular hydrogen bonds, O1—H1···N1 and N1—H1A···O1, respectively, resulting in a R^4^_4_(8) ring motif (Bernstein *et al.*, 1995) about inversion centers, which plays an important role in stabilizing the crystal structure. The R^4^_4_(8) motif units are further linked into a one-dimentional chain along the *b* axis (Table 1 and Fig. 2).

### Experimental

The commercial 2-((4-chlorophenylamino)methyl)phenol (3 mmol) was dissolved in water/EtOH (1:1 *v*/*v*) solution. The solvent was slowly evaporated in air affording colourless block-shaped crystals of the title compound suitable for X-ray analysis.

### Refinement

The H atoms were fixed geometrically and treated as riding with N–H = 0.89), O–H = 0.82 and C–H = 0.93 and 0.97 Å for aryl and methylene H-atoms, respectively, in riding mode on their parent atoms with *U_iso_*(H) = 1.2*U_eq_*(C/N/O).

### Crystal data

**Table:**

C_13_H_12_ClNO	*Z* = 2
*M**_r_* = 233.69	*F*(000) = 244
Triclinic, *P*1	*D*_x_ = 1.345 Mg m^−^^3^
Hall symbol: -P 1	Mo *K*α radiation, λ = 0.71073 Å
*a* = 5.5842 (11) Å	Cell parameters from 2637 reflections
*b* = 7.9485 (16) Å	θ = 3.2–27.5°
*c* = 13.023 (3) Å	µ = 0.31 mm^−^^1^
α = 86.87 (3)°	*T* = 298 K
β = 89.12 (3)°	Block, colorless
γ = 88.65 (3)°	0.10 × 0.03 × 0.03 mm
*V* = 577.0 (2) Å^3^	

### Data collection

**Table:**

Mercury2 (2x2 bin mode) diffractometer	2637 independent reflections
Radiation source: fine-focus sealed tube	1383 reflections with *I* > 2σ(*I*)
graphite	*R*_int_ = 0.061
Detector resolution: 13.6612 pixels mm^-1^	θ_max_ = 27.5°, θ_min_ = 3.1°
CCD profile fitting scans	*h* = −7→7
Absorption correction: multi-scan (*CrystalClear*; Rigaku, 2005)	*k* = −10→10
*T*_min_ = 0.910, *T*_max_ = 1.000	*l* = −16→16
5968 measured reflections	

### Refinement

**Table:**

Refinement on *F*^2^	Primary atom site location: structure-invariant direct methods
Least-squares matrix: full	Secondary atom site location: difference Fourier map
*R*[*F*^2^ > 2σ(*F*^2^)] = 0.068	Hydrogen site location: inferred from neighbouring sites
*wR*(*F*^2^) = 0.187	H-atom parameters constrained
*S* = 0.98	*w* = 1/[σ^2^(*F*_o_^2^) + (0.082*P*)^2^] where *P* = (*F*_o_^2^ + 2*F*_c_^2^)/3
2637 reflections	(Δ/σ)_max_ < 0.001
145 parameters	Δρ_max_ = 0.22 e Å^−^^3^
0 restraints	Δρ_min_ = −0.25 e Å^−^^3^

### Special details

**Table:**

Geometry. All esds (except the esd in the dihedral angle between two l.s. planes) are estimated using the full covariance matrix. The cell esds are taken into account individually in the estimation of esds in distances, angles and torsion angles; correlations between esds in cell parameters are only used when they are defined by crystal symmetry. An approximate (isotropic) treatment of cell esds is used for estimating esds involving l.s. planes.
Refinement. Refinement of F^2^ against ALL reflections. The weighted R-factor wR and goodness of fit S are based on F^2^, conventional R-factors R are based on F, with F set to zero for negative F^2^. The threshold expression of F^2^ > 2sigma(F^2^) is used only for calculating R-factors(gt) etc. and is not relevant to the choice of reflections for refinement. R-factors based on F^2^ are statistically about twice as large as those based on F, and R- factors based on ALL data will be even larger.

### Fractional atomic coordinates and isotropic or equivalent isotropic displacement parameters (Å^2^)

**Table:**

	*x*	*y*	*z*	*U*_iso_*/*U*_eq_	
Cl1	0.3943 (2)	0.26250 (15)	−0.02082 (7)	0.1028 (5)	
O1	1.2067 (3)	0.3735 (2)	0.53925 (13)	0.0478 (5)	
H1	1.2487	0.4639	0.5596	0.057*	
N1	0.6595 (4)	0.3048 (2)	0.41481 (16)	0.0425 (6)	
H1A	0.5273	0.3047	0.4539	0.051*	
C1	1.0518 (4)	0.2978 (3)	0.60896 (19)	0.0394 (6)	
C6	0.8691 (4)	0.2033 (3)	0.5715 (2)	0.0430 (7)	
C8	0.5997 (4)	0.2890 (3)	0.3104 (2)	0.0420 (6)	
C2	1.0819 (5)	0.3070 (3)	0.7143 (2)	0.0503 (7)	
H2A	1.2081	0.3667	0.7391	0.060*	
C7	0.8410 (5)	0.1848 (3)	0.4587 (2)	0.0499 (7)	
H7A	0.9936	0.2038	0.4239	0.060*	
H7B	0.7942	0.0706	0.4472	0.060*	
C5	0.7158 (5)	0.1240 (4)	0.6422 (3)	0.0593 (8)	
H5A	0.5925	0.0603	0.6186	0.071*	
C13	0.3944 (5)	0.3687 (4)	0.2737 (2)	0.0519 (7)	
H13A	0.2966	0.4278	0.3183	0.062*	
C9	0.7429 (5)	0.2015 (4)	0.2432 (2)	0.0544 (8)	
H9A	0.8824	0.1468	0.2666	0.065*	
C11	0.4753 (6)	0.2735 (4)	0.1065 (2)	0.0612 (8)	
C12	0.3308 (6)	0.3628 (4)	0.1723 (2)	0.0620 (8)	
H12A	0.1924	0.4182	0.1484	0.074*	
C3	0.9239 (5)	0.2273 (4)	0.7814 (2)	0.0588 (8)	
H3A	0.9423	0.2353	0.8518	0.071*	
C10	0.6794 (6)	0.1947 (4)	0.1411 (2)	0.0642 (9)	
H10A	0.7768	0.1361	0.0961	0.077*	
C4	0.7407 (6)	0.1367 (4)	0.7467 (2)	0.0632 (9)	
H4A	0.6338	0.0841	0.7929	0.076*	

### Atomic displacement parameters (Å^2^)

**Table:**

	*U*^11^	*U*^22^	*U*^33^	*U*^12^	*U*^13^	*U*^23^
Cl1	0.1312 (10)	0.1295 (10)	0.0494 (6)	−0.0067 (7)	−0.0159 (6)	−0.0148 (6)
O1	0.0486 (11)	0.0435 (11)	0.0524 (12)	−0.0078 (9)	0.0067 (9)	−0.0115 (9)
N1	0.0386 (12)	0.0443 (13)	0.0451 (13)	0.0034 (10)	0.0031 (10)	−0.0089 (10)
C1	0.0347 (13)	0.0364 (14)	0.0470 (16)	0.0056 (11)	0.0013 (12)	−0.0042 (12)
C6	0.0387 (14)	0.0374 (14)	0.0533 (17)	0.0010 (12)	−0.0030 (12)	−0.0050 (13)
C8	0.0392 (14)	0.0426 (15)	0.0447 (16)	−0.0073 (12)	0.0031 (12)	−0.0065 (12)
C2	0.0465 (16)	0.0529 (18)	0.0519 (18)	0.0009 (13)	−0.0075 (13)	−0.0044 (14)
C7	0.0443 (15)	0.0446 (16)	0.0619 (19)	0.0037 (13)	−0.0050 (13)	−0.0134 (14)
C5	0.0491 (17)	0.0526 (18)	0.077 (2)	−0.0091 (14)	−0.0073 (16)	−0.0022 (16)
C13	0.0522 (17)	0.0561 (18)	0.0474 (17)	0.0055 (14)	0.0029 (13)	−0.0063 (14)
C9	0.0459 (16)	0.0616 (18)	0.0575 (19)	0.0015 (14)	0.0018 (14)	−0.0215 (15)
C11	0.069 (2)	0.068 (2)	0.0470 (18)	−0.0101 (18)	−0.0010 (16)	−0.0071 (16)
C12	0.0618 (19)	0.069 (2)	0.0548 (19)	0.0018 (16)	−0.0088 (15)	0.0042 (16)
C3	0.067 (2)	0.064 (2)	0.0447 (18)	0.0054 (17)	−0.0007 (15)	0.0034 (15)
C10	0.068 (2)	0.070 (2)	0.057 (2)	−0.0048 (17)	0.0071 (16)	−0.0235 (17)
C4	0.062 (2)	0.066 (2)	0.059 (2)	−0.0058 (17)	0.0084 (16)	0.0117 (17)

### Geometric parameters (Å, °)

**Table:**

Cl1—C11	1.733 (3)	C7—H7B	0.9700
O1—C1	1.369 (3)	C5—C4	1.380 (4)
O1—H1	0.8202	C5—H5A	0.9300
N1—C8	1.417 (3)	C13—C12	1.376 (4)
N1—C7	1.476 (3)	C13—H13A	0.9300
N1—H1A	0.8898	C9—C10	1.386 (4)
C1—C2	1.390 (3)	C9—H9A	0.9300
C1—C6	1.391 (4)	C11—C10	1.358 (4)
C6—C5	1.384 (4)	C11—C12	1.381 (4)
C6—C7	1.495 (4)	C12—H12A	0.9300
C8—C13	1.376 (4)	C3—C4	1.361 (4)
C8—C9	1.382 (4)	C3—H3A	0.9300
C2—C3	1.374 (4)	C10—H10A	0.9300
C2—H2A	0.9300	C4—H4A	0.9300
C7—H7A	0.9700		
			
C1—O1—H1	110.1	C4—C5—H5A	119.1
C8—N1—C7	117.0 (2)	C6—C5—H5A	119.1
C8—N1—H1A	110.1	C12—C13—C8	121.4 (3)
C7—N1—H1A	110.7	C12—C13—H13A	119.3
O1—C1—C2	121.5 (2)	C8—C13—H13A	119.3
O1—C1—C6	117.9 (2)	C8—C9—C10	120.2 (3)
C2—C1—C6	120.5 (2)	C8—C9—H9A	119.9
C5—C6—C1	117.8 (3)	C10—C9—H9A	119.9
C5—C6—C7	120.8 (2)	C10—C11—C12	120.4 (3)
C1—C6—C7	121.3 (2)	C10—C11—Cl1	120.1 (3)
C13—C8—C9	118.6 (3)	C12—C11—Cl1	119.5 (3)
C13—C8—N1	118.6 (2)	C13—C12—C11	119.1 (3)
C9—C8—N1	122.7 (3)	C13—C12—H12A	120.4
C3—C2—C1	119.5 (3)	C11—C12—H12A	120.4
C3—C2—H2A	120.3	C4—C3—C2	121.2 (3)
C1—C2—H2A	120.3	C4—C3—H3A	119.4
N1—C7—C6	111.5 (2)	C2—C3—H3A	119.4
N1—C7—H7A	109.3	C11—C10—C9	120.2 (3)
C6—C7—H7A	109.3	C11—C10—H10A	119.9
N1—C7—H7B	109.3	C9—C10—H10A	119.9
C6—C7—H7B	109.3	C3—C4—C5	119.1 (3)
H7A—C7—H7B	108.0	C3—C4—H4A	120.5
C4—C5—C6	121.9 (3)	C5—C4—H4A	120.5

### Hydrogen-bond geometry (Å, °)

**Table:**

*D*—H···*A*	*D*—H	H···*A*	*D*···*A*	*D*—H···*A*
O1—H1···N1^i^	0.82	1.97	2.780 (3)	171
N1—H1A···O1^ii^	0.89	2.17	3.037 (3)	165

Symmetry codes: (i) −*x*+2, −*y*+1, −*z*+1; (ii) *x*−1, *y*, *z*.

## References

[bb1] Bernstein, J., Davis, R. E., Shimoni, L. & Chang, N.-L. (1995). *Angew. Chem. Int. Ed. Engl.* **34**, 1555–1573.

[bb2] Fu, D.-W., Ge, J.-Z., Dai, J., Ye, H.-Y. & Qu, Z.-R. (2009). *Inorg. Chem. Commun.* **12**, 994–997.

[bb3] Fu, D.-W., Song, Y.-M., Wang, G.-X., Ye, Q., Xiong, R.-G., Akutagawa, T., Nakamura, T., Chan, P. W. H. & Huang, S. P. D. (2007). *J. Am. Chem. Soc.* **129**, 5346–5347. 10.1021/ja0701816 17428055

[bb4] Fu, D.-W. & Xiong, R.-G. (2008). *Dalton Trans.* **30**, 3946–3948. 10.1039/b806255b 18648695

[bb5] Fu, D.-W., Zhang, W. & Xiong, R.-G. (2008). *Cryst. Growth Des.* **8**, 3461–3464.

[bb6] Rigaku (2005). *CrystalClear* Rigaku Corporation, Tokyo, Japan.

[bb7] Sheldrick, G. M. (2008). *Acta Cryst.* A**64**, 112–122. 10.1107/S0108767307043930 18156677

